# Surgicel™ application in intracranial hemorrhage surgery contributed to giant-cell granuloma in a patient with hypertension: case report and review of the literature

**DOI:** 10.1186/1477-7819-12-101

**Published:** 2014-04-21

**Authors:** Bowen Lin, Hongfa Yang, Mengzhao Cui, Ye Li, Jinlu Yu

**Affiliations:** 1 Department of Neurosurgery, First Hospital of Jilin University, 71 Xinmin Avenue, Changchun 130021, China; 2 Department of Neurosurgery, Jilin Central Hospital, 4 Nanjing Avenue, Jilin Nanjing 130012, PR China; 3 Department of Radiology, First Hospital of Jilin University, 71 Xinmin Avenue, Changchun 130021, China

## Abstract

**Background:**

Surgicel™ is an oxidized cellulose preparation that is widely applied in neurosurgery due to its hemostatic effect and good tissue compatibility. Tumor-like lesions induced by Surgicel® application in cerebral surgery have been rarely reported, especially for intracranial hemorrhage debridement surgery in patients with hypertension.

**Case presentation:**

This case report describes a rare case in which Surgicel™ application led to a foreign body reaction, contributing to the development of an intracranial giant-cell granuloma. A 49-year-old female hypertensive patient was diagnosed with intracranial hemorrhage. She was treated with debridement surgery that employed Surgicel™ application. Although a satisfactory hemostatic effect was achieved, the patient was diagnosed with epilepsy 6 months later. Subsequent magnetic resonance imaging revealed an intracranial space-occupying lesion. After undergoing *en bloc* resection of the lesion, the patient was diagnosed with a Surgicel™-related intracranial giant-cell granuloma by histopathology.

**Conclusions:**

Application of Surgicel™ during intracranial hemorrhage debridement surgery may be associated with a risk of granuloma development due to formation of a tumor-like space-occupying lesion in the surgery bed. Even a low risk of tumor development implies a need for caution when applying Surgicel™, especially when solely used to achieve a hemostatic effect.

## Background

In recent years, absorbable hemostatic materials have emerged as standard tools in neurosurgery. Covering the neurosurgical bed with hemostatic material helps to prevent hematoma due to wound bleeding. As one of the most widely applied absorbable hemostatic materials, Surgicel™ (Ethicon, Inc., a Johnson & Johnson company; Somerville, NJ) is an oxidized cellulose preparation manufactured by Ethicon Inc. of Johnson & Johnson Medical Limited. Extensive clinical evidence has shown that the application of Surgicel™ during cerebral surgery rapidly promotes blood clotting and effectively controls bleeding. Moreover, Surgicel™ has been demonstrated to display superior tissue compatibility compared to other resorbable hemostatic agents [1-3].

A few cases of Surgicel™-induced foreign body reactions have been reported, all of which involved organs outside the nervous system; specifically, giant cell granulomas developed in surgical beds of the cardiac, renal, and adrenal gland tissues [4-6]. To the best of our knowledge, no previous report in the literature has described Surgicel™-induced tumor-like lesions after hemorrhage debridement surgery in hypertensive intracerebral hemorrhage. Herein, we describe a case of giant-cell granuloma in the surgical bed following Surgicel™ application in hemorrhage debridement surgery.

## Case presentation

**Figure 1:**
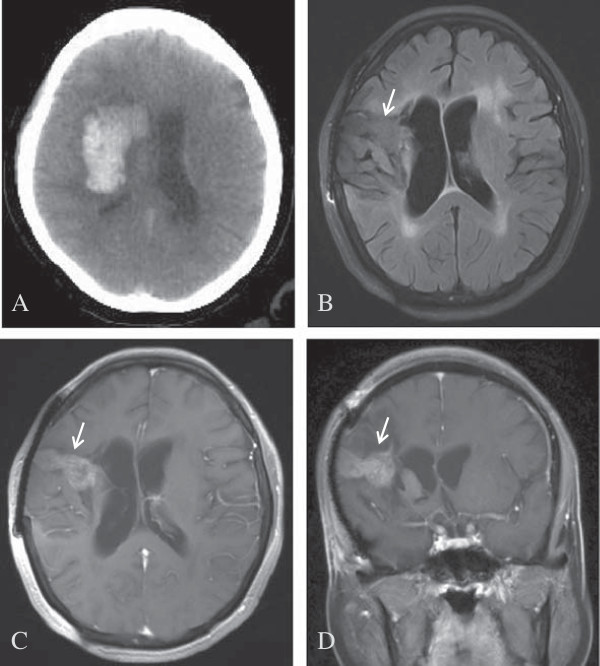
**Images taken before and after surgery. (A)** Computed tomography (CT) scan showing a region with high-intensity hemorrhage in the right basal ganglia. **(B)** Magnetic resonance imaging (MRI) results showing irregularly shaped lesions in the right frontal temporal lobe and basal ganglia, with mixed signal and dark fluid with low or higher signal. Lesion size was 1.7 cm × 3.7 cm. **(C-D)** Lesion observed after strengthening the signal (arrow).

A 49-year-old female with a 5-year history of hypertension was hospitalized for grand mal seizures. Six months prior to hospitalization, the patient had been diagnosed with right basal ganglia hemorrhage via computed tomography (CT) scan (Figure 1A) and underwent treatment by hemorrhage debridement and craniotomy decompression surgery. The surgical routine included the application of Surgicel™ to stop bleeding in the surgical bed. The postoperative recovery was unremarkable, and the patient was discharged to resume independent living. Two months after hemorrhage treatment, the patient received a titanium plate to restore the skull structure. Five months after hemorrhage treatment, the patient suffered two grand mal seizures, which featured transient loss of consciousness and limb rigidity, with episodes lasting approximately 5 minutes. Physical examination upon hospital admission showed left upper limb muscle strength of grade II, left lower limb muscle strength of grade IV, and positive left Babinski sign, but no signs of neurological defect.

**Figure 2:**
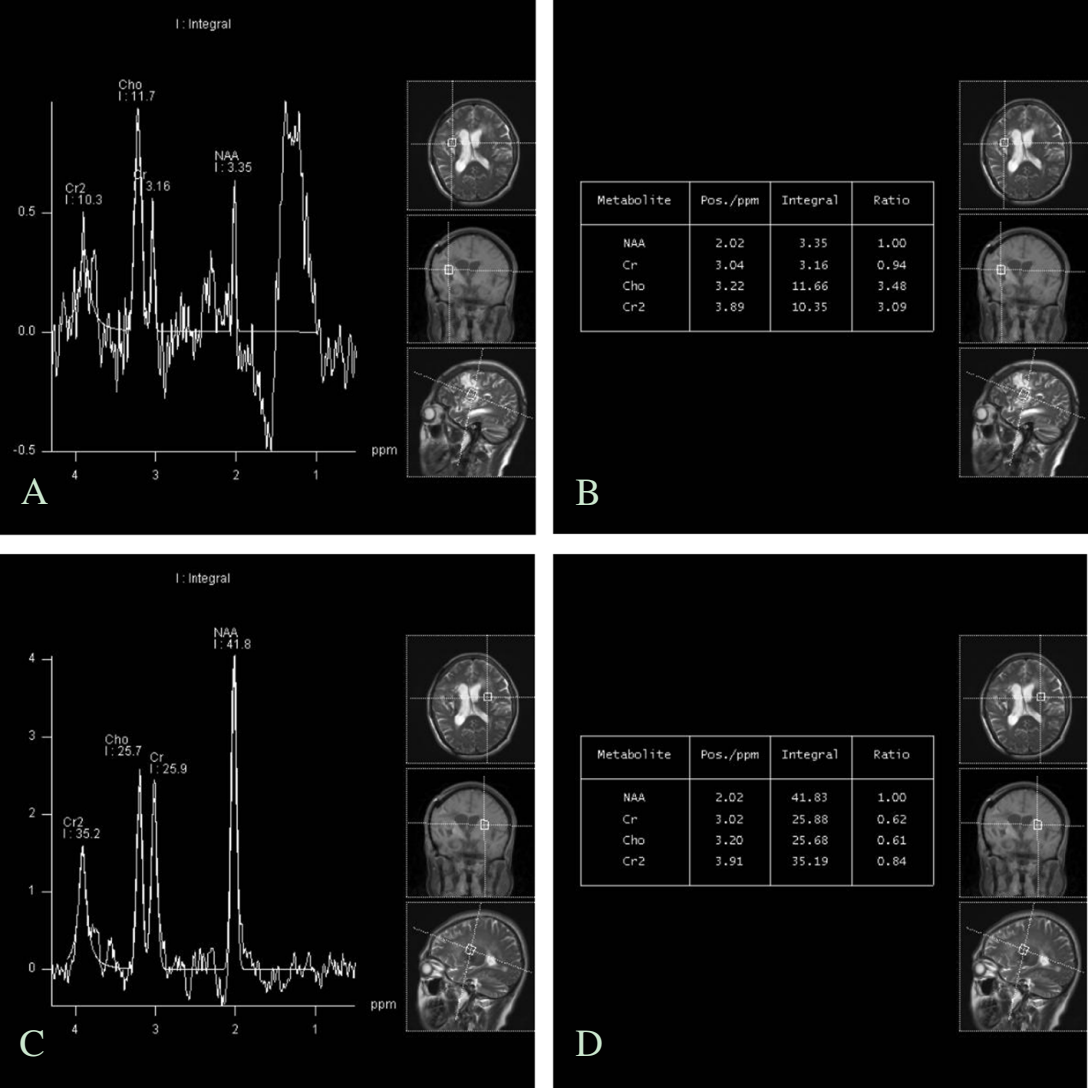
**Magnetic resonance spectroscopy (MRS) analysis. (A)** MRS showed a focally heterogeneous signal intensity and an unstable spectral baseline in the lesion due to signal interference by the liquid and hemosiderin. **(B)** Increased choline (Cho) peak and CHO/N-acetylaspartate (NAA) value were observed. **(C-D)** MRS of the left side was shown.

Magnetic resonance imaging (MRI) studies showed an irregularly shaped lesion in the right frontal temporal lobe and basal ganglia, with a mixed signal (Figure 1B) including reduced signals in T1WI and T2WI and an increased signal indicative of dark fluid. After the signal was strengthened, the MRI-detected lesion measured 1.7 cm × 3.7 cm (Figure 1C, 1D). Magnetic resonance spectroscopy (MRS) studies showed a focal signal of heterogeneous intensity, an unstable spectral baseline in the lesion due to signal interference of liquid and hemosiderin, and an increased choline (Cho) peak and Cho/N-acetylaspartate (NAA) ratio. The presence of abnormal gliosis in the right frontotemporal lesion area led to a diagnosis of suspected tumor (Figure 2).

The space-occupying lesion was surgically resected. The titanium plate was removed, and the frontotemporal lesion and related dural adhesions were visually identified. The gross appearance of the lesion included pale coloration, rough surface, poor blood supply, and clear boundaries. The bottom of the lesion was tightly adhered to the lateral ventricle. Under microscopic guidance, the lesion was completely resected. The patient’s postoperative recovery was unremarkable. A CT scan 5 days after surgery showed complete lesion exenteration.

**Figure 3:**
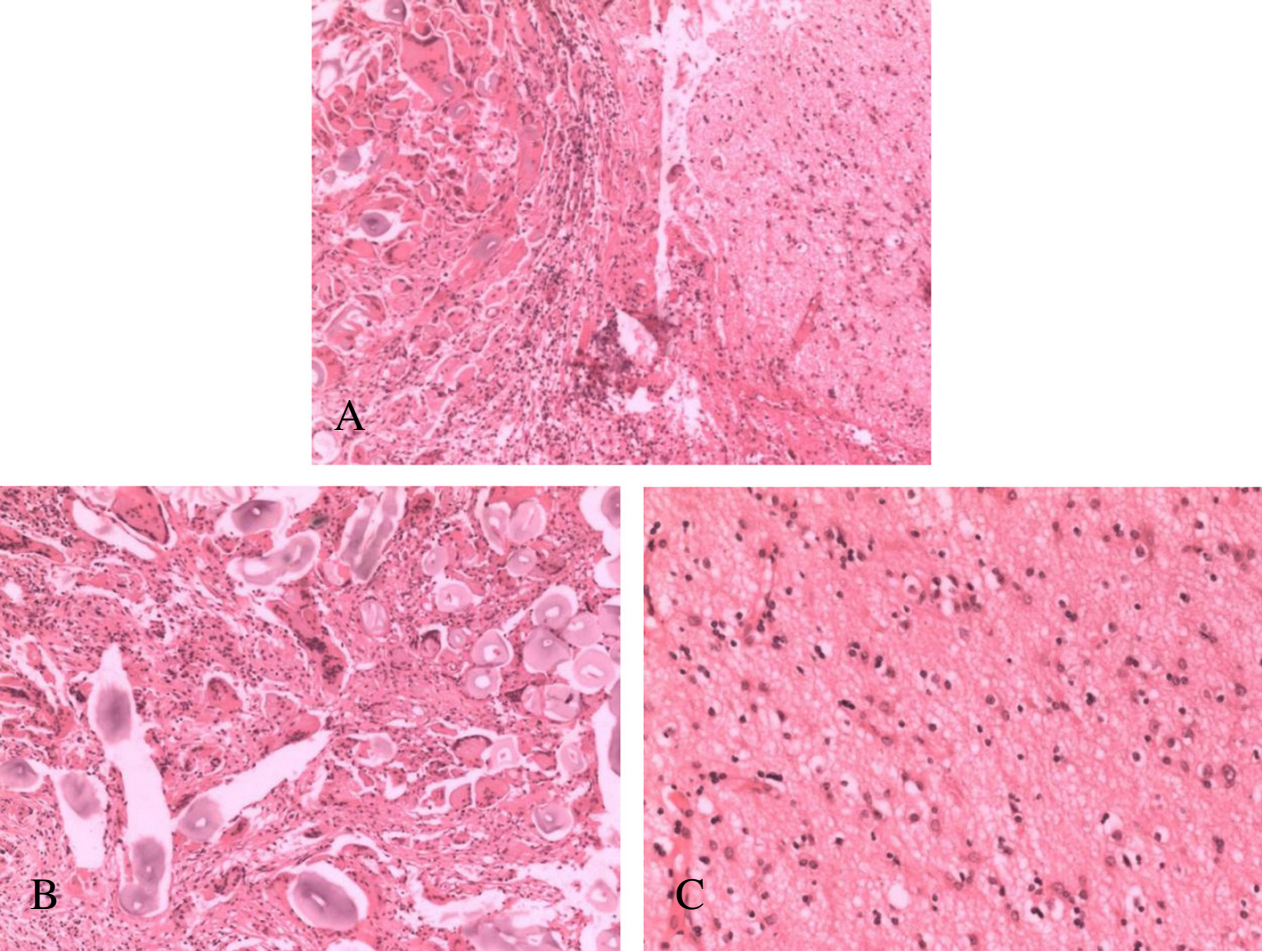
**Histopathologic analysis of resected specimen. (A)** Foreign body and giant cell reaction (left); gliosis (right) (magnification: ×200). **(B)** Foreign body and foam-like cells assembled in the lesion tissue (magnification: ×400). **(C)** Gliosis observed in the lesion (magnification: ×400).

Pathological analysis indicated a foreign body response, as evidenced by substantial amounts of foam-like cells distributed throughout the lesion. The presence of gliosis led to the final diagnosis of a giant-cell granuloma caused by Surgicel™ application (Figure 3). The patient was discharged to home 8 days after surgery. There were no remarkable physical, clinical, or imaging findings at the 6-month follow-up visit.

## Discussion

The formation of surgical beds is unavoidable during neurosurgery, and the normal soft structure of the brain has insufficient strength to support the new vasculature. Traditionally, hemostasis had been achieved during surgery by electrical stimulation. However, this method can cause some vessels to sink into the brain tissue, making coagulation more difficult, or can liquefy the brain tissue, causing irreparable injury [7]. The development of absorbable homeostatic materials to cover the surgery bed overcame the risks associated with electrocoagulation, while effectively achieving control of slight, especially oozing, bleeding. The currently available absorbable homeostatic materials, including gelatin sponge, Surgicel™, and microfiber collagen, are considered suitable for clinical application due to their observed tissue compatibility.

Surgicel™ is an oxidized cellulose polymer, with polyglucuronic glucuronide as its effective functional unit. Blood absorption initiates within 1 day after its application, but complete absorption requires 4 to 8 weeks [8]. Compared to other hemostatic substances, Surgicel™ has an especially high level of tissue compatibility; however, occasional cases of foreign body reactions, such as abscesses, inflammation, and giant-cell granuloma, have been reported with its use [4-6,9,10]. Interestingly, in each case of Surgicel™-induced giant-cell granuloma, the granuloma was initially misdiagnosed as a tumor. For example, Tefik *et al*. reported a case of Surgicel™-related granuloma in a laparoscopic kidney tumor resection surgery that was misdiagnosed as tumor recurrence [9]. Gao *et al*. [10] reported a case of Surgicel™-related granuloma that was misdiagnosed as a tumor 1 month after hysterectomy and right oophorectomy. These previous cases of granuloma formation in organs outside the nervous system were ultimately found to be due to chronic inflammatory reactions. Overactivated giant cells assembled around the Surgicel™, forming the chronic giant-cell granuloma [11].

A similar immune-related mechanism may have contributed, at least in part, to the Surgicel™-related granuloma observed in the brain of our patient. However, a Surgicel™-related granuloma in the brain will have distinctive features from those involving other organs because the blood–brain barrier functions to prohibit giant cell infiltration and assembly in the brain [12]. To address this issue, we reviewed the clinical characteristics of the previously reported Surgicel™-related granulomas, and compared them to those of the granuloma in our patient.

Sandhu *et al*. reported that the clinical manifestations of two Surgicel™-related granulomas occurring after brain tumor resection were similar to those of other intracranial space-occupying lesions [13]. The time to granuloma formation after Surgicel™ application in organs outside the nervous system ranges from 1 to 36 months [10]. The two Surgicel™-related granulomas in brain reported by Sandhu *et al*. formed within 1 and 3 months after surgery, respectively. In contrast, Ito *et al*. reported three cases of Surgicel™-related granuloma formation in brain that occurred within the time span of 13 to 21 months [14]. Hence, the few reported cases of Surgicel™-related granuloma in brain appear to have formed within the same timeline as Surgicel™-related granulomas in other organs outside the nervous system. Consistent with this finding, the Surgicel™-related granuloma case presented herein formed 6 months after surgery for cerebral hemorrhage.

Another intriguing feature of Surgicel™-related granulomas is the fact that they are commonly misdiagnosed as brain tumors, particularly in cases of granuloma forming after cerebral tumor resection. In two previous cases, the Surgicel™-related granuloma manifested as a space-occupying lesion with heterogeneous density by CT scan [13,14] and mixed-signal with surrounding edema by MRI [15]. Such imaging features do not facilitate the ability to distinguish a granuloma from a brain tumor, increasing the risk of misdiagnosis. For our patient, MRS was used for further investigation of the lesion and helped to identify the granuloma as a tumor.

To confirm the MRS-based tumor diagnosis, histopathologic analysis of the resected lesion was performed. Hematoxylin-eosin (HE) staining of the unabsorbed Surgicel™ substance showed mild eosinophil infiltration (indicated by blue or lavender coloration). Because the blood clot around the unabsorbed Surgicel™ was gradually absorbed, the Surgicel™ was left in place. The blood clot appeared in the HE-stained lesion sections as a ‘ghostly’ fiber surrounding the Surgicel™ substance, and no tissue accumulation was observed surrounding the Surgicel™. Notably, numerous multinucleated giant cells were found around these fibers [2]. The pathological hallmarks of this case were consistent with the features of granuloma. Hence, the case was diagnosed as Surgicel™-elicited granuloma. Although the features of Surgicel™-related granuloma are not distinguishable from those of tumor, this case indicates that a tumor-like space-occupying lesion that forms in the surgery bed after Surgicel™ application during non-tumor surgery may have a high risk of becoming a Surgicel™-related granuloma.

## Conclusions

Although low, the risk of developing Surgicel™-related granuloma after surgery indicates a need for caution in applying Surgicel™, especially when it is used solely for the purpose of achieving a hemostatic effect. However, the risk of granuloma may be reduced by removing the unabsorbed Surgicel™ after the hemostatic effect has been achieved, or by reducing the amount of Surgicel™ applied. When a tumor-like space-occupying lesion is observed in the intracranial surgery bed after surgery employing Surgicel, the rare possibility of Surgicel™-related granuloma should be considered.

## Consent

Written informed consent was obtained from the patient for publication of this case report and accompanying images. Copies of the written consent are available for review upon request.

## Abbreviations

Cho: choline; CT: computed tomography; HE: hematoxylin-eosin; MRI: magntic resonance imaging; MRS: magnetic resonance spectroscopy; NAA: N-acetylaspartate.

## Competing interests

The authors declare that they have no competing interests.
